# Supplementary material for: Validity and Reliability of a Smartphone Application for Home Measurement of Four-Meter Gait Speed in Older Adults
Source: Bioengineering (Basel). 2024 Mar 6;11(3):257. doi: 10.3390/bioengineering11030257 (PMC10968134; doi:10.3390/bioengineering11030257)
Supplement: Supplementary file 1 [file bioengineering-11-00257-s001.zip › bioengineering-2855833-supplementary.pdf]

FIGURE CAPTIONS

Figure S1: A visual representation of the study procedure over a span of five days within one week. The study staff were present with the participants for the first and second days ("Supervised"), and the study staff were not present for the third to fifth days ("Unsupervised"). Under the supervised days, the study staff first taught the participants how to use the smartphone App and the participants would then self-administer the smartphone App. At the same time, the study staff captured their walking performance with a video camera and a stopwatch. Under the unsupervised days, the participants self-administered the smartphone App at their home without any feedback, guidance, or assistance.

Figure S2: Example of smartphone-recorded direct cosine matrix (DCM) relative to the earth coordinate system medio-lateral axis of the body during straight walking and a 180° turn. The vertical line represents the start of turning.

Figure S3: Bland Altman plot for 4MGS measured by the smartphone App-based assessment compared to gold standard video-based assessment. The solid lines are the average differences between methods, and the dashed lines are the limit of agreements (± 1.96 SD).

Figure S4: The Passing-Bablok orthogonal regression evaluating the association and the relationship (r: correlation coefficient) between 4MGS measured by the smartphone

App-based assessment and by the stopwatch (A) and from gold standard video-based assessment and from the stopwatch (B). The red dashed line represents the 45-degree line, indicating perfect agreement between two methods. The blue line represents the linear regression line that best fits the data points, and the blue dashed lines indicate the 95% confidence interval.

Figure S5: Bland Altman plot for 4MGS measured by the smartphone App-based assessment compared to the stopwatch (A) and gold standard video-based assessment compared to the stopwatch (B). The solid lines are the average differences between methods, and the dashed lines are the limit of agreements (± 1.96 SD).

Figure. S1

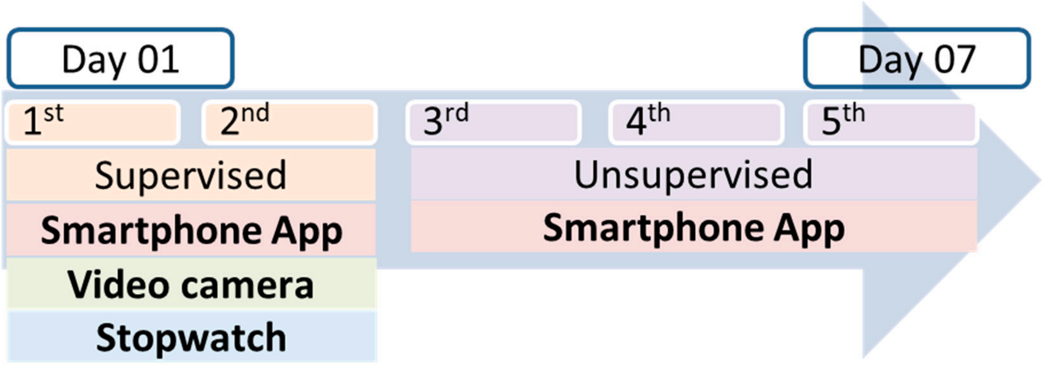

Day 01
Day 07
1st
2nd
3rd
4th
5th
Supervised
Smartphone App
Video camera
Stopwatch
Unsupervised
Smartphone App

Figure. S2

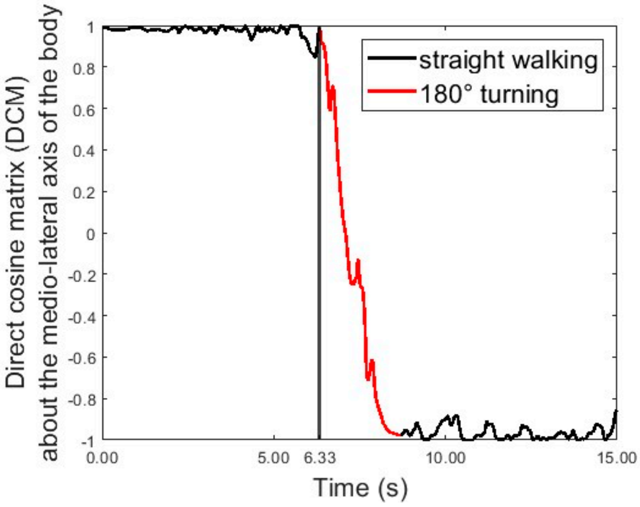
Direct cosine matrix (DCM) about the medio-lateral axis of the body
1
0.8
0.6
0.4
0.2
0
-0.2
-0.4
-0.6
-0.8
-1
0.00
5.00
6.33
10.00
15.00
Time (s)
straight walking
180° turning

Figure. S3

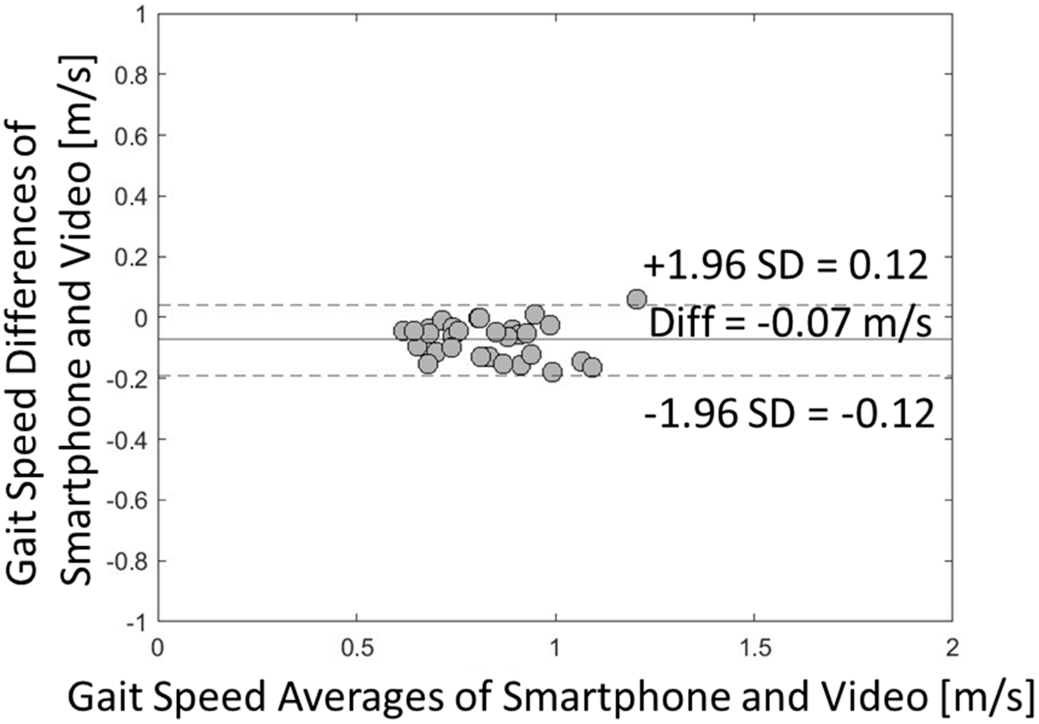
+1.96 SD = 0.12
Diff = -0.07 m/s
-1.96 SD = -0.12
Gait Speed Differences of Smartphone and Video [m/s]
Gait Speed Averages of Smartphone and Video [m/s]
1
0.8
0.6
0.4
0.2
0
-0.2
-0.4
-0.6
-0.8
-1
0
0.5
1
1.5
2

Figure. S4

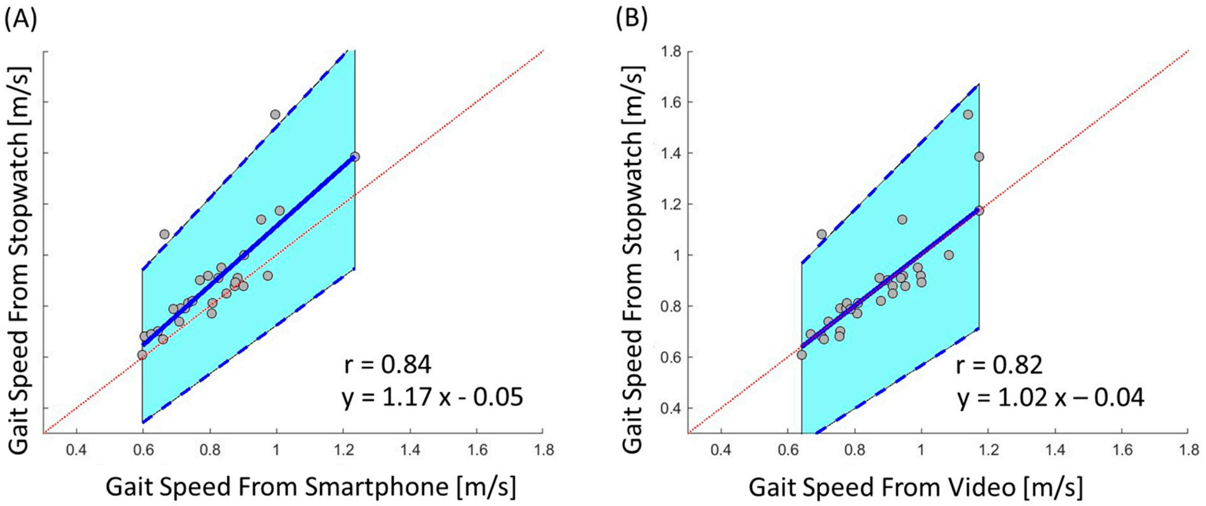

(A)
Gait Speed From Stopwatch [m/s]
r = 0.84
y = 1.17 x - 0.05
0.4
0.6
0.8
1
1.2
1.4
1.6
1.8
Gait Speed From Smartphone [m/s]
(B)
1.8
1.6
1.4
1.2
1
0.8
0.6
0.4
Gait Speed From Stopwatch [m/s]
r = 0.82
y = 1.02 x – 0.04
0.4
0.6
0.8
1
1.2
1.4
1.6
1.8
Gait Speed From Video [m/s]

Figure. S5

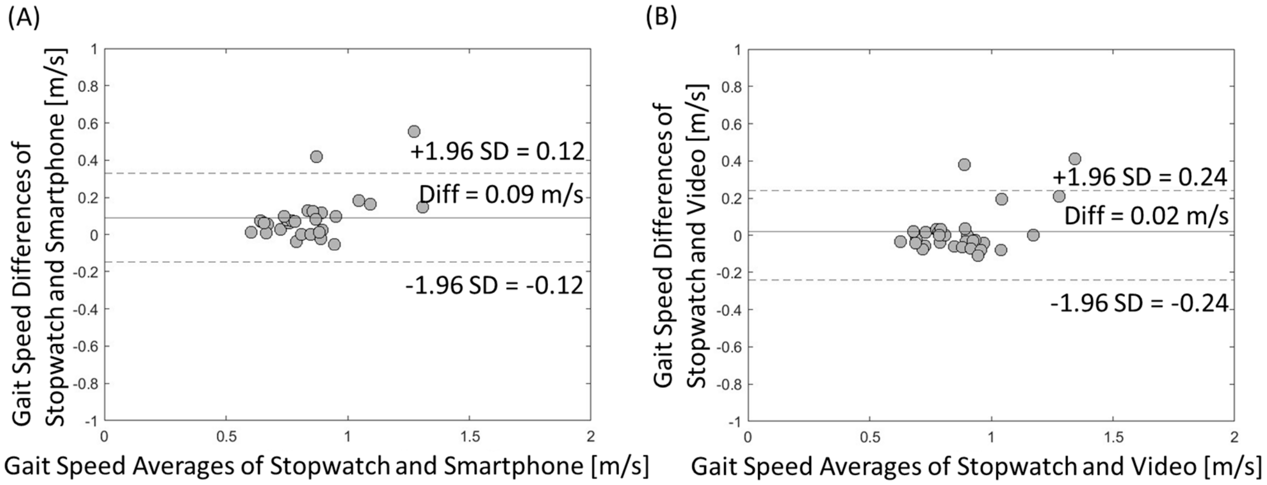
(A)
+1.96 SD = 0.12
Diff = 0.09 m/s
-1.96 SD = -0.12
Gait Speed Differences of Stopwatch and Smartphone [m/s]
Gait Speed Averages of Stopwatch and Smartphone [m/s]
(B)
+1.96 SD = 0.24
Diff = 0.02 m/s
-1.96 SD = -0.24
Gait Speed Differences of Stopwatch and Video [m/s]
Gait Speed Averages of Stopwatch and Video [m/s]
